# Principles of Pathology and Practice of Physic

**Published:** 1836

**Authors:** 


### REVIEWS AND BIBLIOGRAPHICAL NOTICES.

### ARTICLE. III. — PRINCIPLES OF PATHOLOGY AND PRACTICE OF PHYSIC.

*Principles of Pathology and Practice of Physic.*—By JOHN MACKINTOSH, M. D., lecturer on the Practice of Physic in Edinburgh, from the last London edition, with notes and additions by Samuel George Morton, M. D., etc., etc. Two volumes, pp. 462 and 509. Key and Biddle, Phil. 1836.

This is the third edition of this valuable work, which has been variously amended by the author, since the first edition was issued from the press. Dr. Morton is known to our readers as a cultivated, industrious and useful medical writer. The contributions of his pen to the present work are characterized by research, and sedateness of reflection, and are useful additions to the original matter. Our object in the present instance will be to present an analysis of the work, occasionally offering suggestions of our own, either to elucidate the text, or 'to' make kown our own views on points in which we may differ from the author, or American editor.

Part 1.—General History of Inflammation and Fevers, with the Pathology and Treatment of Individual Fevers.

"*Chapter* 1.—Of Inflammation." Under this head the author discusses the general doctrines, causes, phenomena, and effects of inflammation. He has traversed rapidly the often trodden ground of former opinions, once so celebrated, entertained on this point, and then advances his own particular views. The latter we shall give the reader in the author's own words.

"It appears to me, that the view taken by Mr. Syme, in an essay on inflammation, (Edinburgh Medical and Surgical Journal, vol. 30, p. 316) is the most philosophical. He thinks that too much attention has been directed to the obvious signs of inflammation, viz: redness, heat, swelling and pain, and too little bestowed on the *altered functions of the part.* Mr. Syme justly thinks that if this remarkable character of inflammation had been kept in mind, pathologists would hardly have spent so much labor in disputing about contraction and dilatation of the vessels, since it is obvious, that mere difference of capacity, though it might, to a certain extent, account for the redness and swelling, could never enable us to explain the alteration of function, any more than a knowledge of the size of capillary vessels could instruct us as to the mode in which their secretions, etc., are performed in health. And he maintains that redness and swelling ought to be secondary considerations in the investigation of the inflammatory state, in comparison with the grand distinguishing character of altered functions."

"Three points seem to have been much overlooked by writers on inflammation. First, The influence of the nervous system. Secondly, The changes in the qualities of the blood itself; and, Thirdly, The disordered functions of the capillaries. I have performed experiments on horses, which prove most satisfactorily the influence which the nerves have, even in chronic inflammation. It is well known that these animals are very liable to inflammation in the foot, from different causes; and I have seen horses who had been lame for months, cured by dividing the nerves above the fetlock joint; the effect being sometimes instantaneous, and occasionally permanent."

"With regard to the second point, there can be no doubt that the blood in the part affected becomes diseased; the red particles cease to be observed, and the blood assumes a flocculent appearance, becoming darker and darker, and the vessels become in some degree obstructed. It is not improbable that this change on the blood may be found to depend partly, if not principally, upon the cessation of nutrition and exhalation, and at the same time a stop being put to the conversion of arterial into venous blood."

"The essence of inflammation, partly consists in more blood entering by the arteries than can escape by the veins, or than can be made use of when the part is in a state of health, when its functions are actively performed; the consequence is an accumulation of blood, or congestion and effusion from partial obstruction; and it is, I imagine, this degree of obstruction which produces the throbbing. The vessels of the inflamed part are greatly dilated, and the number which contains red blood is greatly increased."

"It must be confessed, that in inflammation there is much undiscovered. Physiologists have to settle several disputed points in the doctrines of the circulation; and anatomists have to discover a great deal regarding the anatomy and physiology of the nervous system, before pathologists can be expected to advance their part of the science of medicine in any remarkable degree." p. 14–15. We perfectly coincide with the author in his declaration of the ambiguities which yet environ the subject of inflammation. There seems to be a disposition in the medical world to regard inflammation in too arbitrary and abstract a manner—as a simple augmented condition of action in the parts affected, instead of viewing it as series of morbid processes going on in the nervous and vascular portions of the organism. Inflammation is not a single or simple act of the vascular apparatus of the part affected with heat, pain, redness and tumefaction, but it is essentially constituted of several distinct consecutive acts. 1st. There is a state of incubation—or the forming stage—one of modified sensibility and irritability; 2dly. There is active congestion—or accumulation of blood; and 3dly, There is altered conditions of the blood itself—Such as a breaking down of the red globules, interstitial deposit, and effusion of coagulable lymph. The able works of Gendrim and Kaltenbrunner have well enforced and illustrated this very important topic of medical investigation. To them we would refer for a more detailed elucidation of this question. We proceed to give additional extracts from our author.

"Phenomenon of Inflammation.—External inflammation is characterized by redness, swelling heat, and pain. All these, taken together, leave no doubt as to the existence of inflammation. In this respect, surgeons have the advantage of physicians. They can see and feel the part affected, in addition to the power of judging from constitutional symptoms, and the account the patient gives of his own sensation. Whereas, in physic, we have greater difficulties to encounter in forming a diagnosis. We observe local and constitutional symptoms also; but it does not always follow, because there are dyspnœa and fever, that the lungs are inflamed; the disease may be inflammation of the pericardium. There may be violent vomiting, tenderness in the epigastrium, thirst, with more or less fever, while the disease is in the head. There may be some local and constitutional disturbance, without the existence of the slightest degree of inflammation, merely from a neuralgic affection of some tissue or organ, or from impeded function of some viscus. During life we cannot see the state of internal organs to ascertain whether they are red and swollen; and a sensation of heat, pain, and fever may exist without the least inflammatory action. It will be proved in a subsequent part of this work, that the pulse cannot be depended on. With respect to buffy blood, it may exist without actual inflammation; and, in inflammatory complaints, the blood does not always yield it. The shape of the dish modifies this appearance, so does the manner in which the blood flows from the vein. Mental agitation and fatigue produce the buffy coat. Sometimes it does not appear on the blood till the patient has been largely and repeatedly bled. I am inclined to place considerable dependence, however, on the buffy blood, *taken in connection with other circumstances*, particularly when the surface is also concave, or "cupped," as it has been termed, and when the quantity of serum is proportionably large.

"It has often occurred to me to see dissections where great destruction of vital organs had taken place from inflammation, and yet there had been little or no pain complained of during life. Nay, I have seen instances of inflammation of the pleura to such a degree as to occasion death, where the symptoms were too slight to direct the medical attendants to the true seat of the disease.

"I feel convinced that no pathological physician will join Dr. Gregory, a modern writer on the practice of physic, in the following curious dogmas: *Delirium marks inflammation of the brain; impatience of light, opthalmia; hoarseness, inflammation of the larynx; and dyspnœa, that of the lungs.* The practice of physic would indeed be simple and certain, were these things true. The uncertainty of the pulse has been already mentioned. Inflammation may be going on towards a fatal termination in an important organ, without any febrile movement. This was noticed long ago by Morgagni, Valsalva, and others; and it led them too hastily to conclude, that mortification of internal organs occasionally took place without the previous existence of inflammatory action." p. 21.

We have presented the reader in the above extracts a condensed view of the author's views on this very important point. A perspicuous and practical conception of this most interesting pathological question is of vital consequencee to the scientific physician. The separate consideration of various inflammatory diseases will come up for our inquiry in the further analysis of the work.

The next general point treated of by the author is fever, the history of its general doctrines being first considered.

After a brief examination of the once illustrious and popular theories of Galen, and Boerhave, the author criticises with some minuteness of remark, the opinions of Cullen on this point. We need not quote his reflections on this eminent teacher's doctrine of fever—there has been an immense accumulation of critical annotation on this head since the days of that erratic "son of genius and misfortune," Brown, to this period. Brown Darwin, and Lutterbuck are succinctly noticed—Broussais receives a commendatory, although, superficial reference from the author, but an expanded and luminous exposition of his peculiar febrile pathology is given by the American editor. The editor enters upon a rather protracted analysis of the laws of irritation, as taught in the physiological school, after which he proceeds to consider Broussais' theory of fever, founded upon them. "According to this hypothesis, all fevers owe their origin to a [illegible]ral irritation or inflammation, by the reaction of which up[illegible] the other organs, through the medium of the sympathies, that group of phenomena is produced which is called febrile disturbance. All that class of fevers commonly termed idiopathic, together with certain forms of traumatic fever are attributed to an inflammatory condition of the mucus membrane of the stomach and bowels, and are therefore denominated *gastro enterites*." Boisseau, an ardent admirer and zealous follower of Broussais in some of the great outlines of his scheme of explanation, withholds his assent from the proposition that all fevers originate from local irritation of the stomach and bowels, though he strenuously urges that they arise from some point of inflammatory action. The following sentences contain the authors views. "First, Fevers may depend on inflammation of an acute, but more frequently of a subacute nature of some organ or tissue of the body. If the inflammation be acute, the febrile symptoms will be correspondently high; but if subacute, they will assume a slighter form.

Secondly. Fevers very often depend upon mere functional derangement of some organ, having as yet no connexion with inflammation.

Thirdly. Fevers sometimes depend on the mere loss of balance in the circulation producing local congestions; fevers arising from these last two causes are generally called idiopathic.

"My ideas of fever may be summed up in the words of Dr. Fordyce, one of the best and most original writers upon the subject. A fever, says he, is a disease that affects the whole system, affects the head, the trunk of the body, and the extremities; it affects the circulation, the absorption and the nervous system; it affects the skin, the muscular fibres and the membranes; it affects the body, and affects likewise the mind. It is, therefore, a disease of the whole system in every kind of sense. *It does not, however, affect the various parts of the system uniformly and equally; but, on the contrary, sometimes one part is much affected in proportion to the affection of another part.* Dissertation on simple fever, part 1, p. 27."

"Division of Fevers."—The following arrangement of fevers the author gives as his own, in preference to any other.

1st. Intermittent fever.

2d. Remittent or yellow fever; infantile remittent.

3d. Continued fever, subdivided into four orders, viz. Fever from functional derangement—from inflammation—from congestion. A mixed form of fever between these three last, but in which congestion predominates, commonly denominated typhus or synochus. 4th. Hectic fever. 5th. Fevers attended with eruptions, subdivided as follows: Scarlet fever, measles, small pox, modified chicken pox, miliary fever, roseola, urticaria. 6th. The Plague." p. 56.

"General description of the phenomena of fevers."—The author reiterates the common observation in reference to the want of a true pathognomonic symptom in fever. We conceive fever to be a sort of epitome of disease—for in a legitimate seizure of idiopathic fever every organ and function of the body is liable to be affected by the disturbing presence of the febrile action. Hence the impracticability of detaching any one symptom from the concatenated train of phenomena, and of making that symptom peculiar and characteristic of the morbid action. The manifestations of the febrile state vary every day in the course of the disease. The accession of fever may be marked by a chill, or there may be a total absence of chilliness; the increase of the fever may be gradual, or rapid—its declensions may be denoted by the breaking out of a profuse perspiration—or there may be no such secretory eruption—and the last stage—that of collapse—may likewise vary in its mode of accession.

In fever the functions of every organ are more or less disturbed, so that there is the best proof of a universal disorder, and the appearances so frequently seen on dissection warrant this inference. True it is, that we now and then, on examining the body of an individual, find no decided morbid appearance. This is by no means peculiar to the practice of physic; for, in that of surgery, people sometimes die of capital operations, where there has been no loss of blood, and no organic lesion found upon dissection, to explain the cause of death. They are said to die from the shock; by which term I understand that the principal functions of the body become suddenly impeded to such a degree that life can be no longer carried on. In the same way, in fevers, individuals die before any alteration of structure has taken place; from peculiarity of constitution, they cannot stand the shock produced by the embarrassment of so many organs in the performance of their functions; and farther, many individuals cannot bear the remedies which have been thought necessary for the subduction (subdual) of the disease. p. 58.

The author divides continued fever into several varieties—such as, fever from functional derangement—inflammatory fever—conjestive or *fievres intermittentes pernicienses*, which occurs in Rome and other places, where intermittents prevail, the pathological elucidation of which has been so fully pointed out by M. Bailly, a mixed form of fever—synochus at the commencement terminating in typhus—both of which terms, he conceives, to be objectionable.

In our mode of viewing this interesting subject all the above distinctions are mere theoretical refinements, as far as the true pathological identity of fever is concerned. We regard fever as, at its origin, to be a functional disease; originating in a disturbed state of the nervous system—which rapidly passes on to a condition of morbid action in the secretory organs, and this functional disturbance brings on vascular irregularity, which often terminates in local inflammation. It is very clear that we find many patients laboring under both topical inflammation, and simple functional disturbance who have no regular seizure of fever. There seems another element of the disease wanting, or rather we should say, there seems a total absence of the seminal principle of febrile movement—the modification which the sensibility and irritability of the system has undergone—termed by recent authors of high reputation in the profession—the state of incubation of the disease.

"Causes of Fever."—The causes of fever are marsh, miasm, contagion from human effluvia, and epidemic influence. These causes, together with cold, fear, etc., are called in medical language remote; but I shall continue to employ the terms common and specific. Cullen resolves all remote causes into sedative, in order to support his dogma of debility, etc. Again the author remarks on this point. No one who has attended to this subject can deny the influence of contagion, and the air of marshes, on the human body; but I conceive that too much has been attributed to them, too little to the previous state of the constitution, and also by far too little to the common causes of fever, and to internal irritations." p. 62.

The author considers that contagion rarely operates in the production of fever if the person exposed to its agency, "is in a good state of body and mind. Dr. Gregory used facetiously to assert that contagion might lie frozen for any length of time, and resume its virulence upon being thawed. The Dr.

stated, that he must have been exposed to the influence of contagion some 20 or 30,000 times, without affecting him once."

From personal observation, during a residence in a marshy district, the author has derived the following facts—1st. That women were less liable to the disease than men—blacks than whites—the sober than dissipated men; "and that agues were most prevalent at new and full moon."

The comparative immunity enjoyed by females arises from their domestic habits—their greater regularity of life, and more temperate mode of living. The same obtains in reference to blacks, besides a difference of constitution, which is influential in their favor.

"Moisture, alone, has a great effect in producing disease, and its influence is speedily observed on the mind as well as the body. But moisture alone will not produce intermittent fever: the influence of excessive heat must be superadded, and then there is a rapid evaporation from the earth's surface."

"It will be seen that it is not my intention to deny the existence of some invisible substance suspended in, or mixed with the air of the atmosphere, and which may produce intermittent fever. A fact may be mentioned on this side of the question, which must carry considerable weight with it. It has occurred to me, to see a good deal of intermittent fever in situations far remote from marshes, but in every one instance the individuals had been at some period of their lives in marshy districts; yet it is certainly very strange, that some of them never had a paroxysm during the period of their residence in these places, and not till months, and in some instances, years had elapsed." p. 65.

The author believes that intermittent fever is never contagious; but is of opinion that yellow fever, and that fever which has been termed typhus, in England, "are so, under particular circumstances, in a very high degree." Observation and experience, however, have induced him to conclude that this cause of fever has been very much overrated. We entirely coincide with this last declaration, and are very confident that our author has, with all his reservation on the subject, violated the spirit of a just philosophy in attributing contagiousness in any degree, to either yellow fever, or typhus.

There are very few physicians in the United States who admit the correctness of the author's opinion concerning the contagious property of yellow fever. This question has been put to rest by the elaborate inquiries of the learned Dr. Ch's Caldwell—whose investigations on this interesting question have most triumphantly settled the point. Dr. Rush, with that candor and magnanimity which ever guided him in his noble and illustrious career, has acknowledged his indebtedness to Dr. Caldwell on this subject. Since the removal of the great reformer of American Medicine from his earthly labors to the high rewards of a future state, Professor Caldwell has communicated additional reflections on the question, and other American and European writers have likewise successfully cultivated this part of the great field of medicine.

In speaking of critical days, our author remarks, "Pathagoras first started an opinion respecting critical days, and he had an unlimited belief in the occult powers of certain numbers. Hippocrates seems to have entertained similar opinions, and it is an essential part of the old doctrines of concoction, according to which it was supposed that a separation of the morbific matter had a tendency to take place on one of the critical days, by a discharge from the skin, bowels, kidneys, or blood vessels.

"I have no belief in the influence of critical days, although I admit that the crisis frequently takes place in some of the ways mentioned." "From the time of Hippocrates, it has generally been believed that fevers had a tendency to remit on the 3d, 5th, 7th, 9th, 11th, 14th, 17th, 20th days, and even on the 21st. Many modern physicians have adopted this doctrine; but I doubt much whether it has not proved more injurious than beneficial in the treatment of disease. Often may the physician be seen prescribing a placebo, because the critical day is at hand, when they ought to be actively employed in eradicating the disease. When attending to this point I have often found the calculations made erroneously; and not unfrequently I have seen physicians disagree as to which was the critical day—one calculating from the period when the rigor took place—another from the period when the heat of skin occurred—and I have seen a third calculation made from the time when the patient confined himself to bed." p. 75.

"But it is of little importance whether the doctrine of critical days be true or false, if the physician acts wisely, and neglects nothing which can tend to reduce the diseased action." p. 76.

"Intermittent Fever."—Under this head we find little that is either novel, or very important, till the author discusses the treatment. The cases of pathological anatomy, extracted from Mr. Bailly's work, entitled, Traite' Anatomico-Pathologique des Fievres Intermittentes, Simples et Pernicieuses, published in 1825, have but little inherent weight. They only prove that intermittent fevers occurring in Rome and its unhealthy vicinty, wore, very often, an aspect of great malignancy, and that persons dying of an attack of ague were found affected with inflammation of the brain, or other vital organs. Such complications may occur without proving that there is in intermittent fever, the constant presence of topical inflammation. We have known patients affected for months with ague and fever, who were in the enjoyment of a good appetite and digestion on the intermediate days, and were capable of a vigorous discharge of the functions of life, in the absence of the paroxysm. Such facts clearly demonstrate that intermittents do not owe their existence in the system to local inflammation, however confidently Broussais and his disciples may urge this view upon the profession. In his discussion of the pathology of intermittents, the author advances nothing new, but merely reiterates the belief that congestion is the cause of all the morbid phenomena—how the congestion is brought about, he pretends not to determine, but says, "perhaps the first link in the chain of morbid action may be in the nervous system; there is decided evidence of its being involved from the beginning to the termination of the disease. But as there is nothing to guide us in the investigation, I shall not enter into it." But, contrary to the above, we conceive that there is as much to guide us in our observation of the phenomena as springing, in the estimate we form of their primordial link, from a disturbed state of the nervous system, as from the rather mechanical notion of their origination from congestion. A simple state of retrocession of blood from the vessels of the surface, as may be induced at any time by the cold bath, will not generate the subsequent phenomena of an intermittent. The prior morbid state of the nervous system, is the plastic element at work, in an attack of genuine intermittent; which element of abnormal action reveals its presence and activity by the subsequent symptoms which consecutively flow from it in the development of the seizure. Mr. Brachet grounds his theory of intermittent upon the peculiar views which he takes of the offices and connexions of the two nervous systems, the cerebro-spinal and ganglionic, the primary lesion being in their derangement to the exclusion of irritation, as understood by the physiological school.

As our author entertains some peculiar views of great practical weight upon the great point of the best method of treating intermittents we will let him speak for himself.

"*Treatment of the cold stage.* Bleeding in the cold stage will, in a great majority of instances, cut it short; in fact it will rarely fail in stopping the existing paroxysm, and on many occasions it has prevented a return of the disease to which the patients had been long subject, and by which they were nearly worn out. It is difficult to determine what quantity of blood it will be necessary to draw in any given case; sometimes it requires twenty-four ounces; I have known three ounces suffice, and, in one case, an ounce and a half produced the full effect. The larger the orifice in the vein is made, the greater is the chance of arresting the disease at a small expense of blood: but in many cases, the operation is attended with considerable difficulty, from the convulsive tremors which affect the whole body. I was once successful in arresting the disease by bleeding, in a cold stage which had continued twenty-four hours; but I regard this as an extreme case. The blood sometimes only trickles down the arm, and as the system is relieved, the stream becomes larger and stronger, till at last it springs from the orifice, and frequently before six ounces are taken, the patient will express relief from violent pain in the head and loins, and it will soon be observed that he breathes more freely. The tremors become slighter and slighter, and, by the time a few more ounces are abstracted, they will cease altogether, and with them will vanish the painful sensation of cold. The pulse will be found stronger, and a gentle moisture will be observed on the body. If the patient be properly managed with respect to bed clothes, neither hot nor sweating stage will in general follow."

The author quotes thirty-two cases in which bleeding in the cold stage was eminently beneficial. He answers several ob jections which have been urged against the remedy, and in conclusion of his recommendation of venesection, says, "I wish to impress upon the minds of my readers, that by venesection in the cold stage of intermittents, we stand upon vantage ground, by affording our patients the benefits of the following circumstances.

1st. The injury which in many cases results from the continuance of the venous engorgement, which so constantly leads to organic diseases, is avoided.

2d. The danger proceeding either from the want of sufficient reaction, or from its excess, is also avoided.

3d. The practice prevents debility in a direct manner, by saving the vital fluid.

4th. The chance of a return of the paroxysm is diminished, or if it should recur, the force of the attack will, in general, be weakened; and in that case a most important point will be gained, by affording an opportunity for the administration of other remedies, as bark or arsenic, which might previously have been exhibited in vain.

5th. Experience has also taught me, that bleeding in the cold stage is far more efficacious than bleeding during the hot stage, or in the intervals." p. 126.

Our author has rather, we think, fallaciously urged the propriety of venesection in the cold stage of intermittent fever by the statement of what he terms " a curious and interesting fact"—"that some Persian physicians apply ice to the surface of the body in the cold stage of intermittents, and it is reported with good effect." This is a sad method of proving the judiciousness of any plan of practice—we look upon such loose reasoning, and unprecise proofs, with sorrowful feelings of doubt respecting the whole grounds of discussion. In candor we must state that we have never given the remedy, so highly commended by the author, a trial at all commensurate to the importance attached to it by him. The lancet has been used by us in the hot stage, and during the interval, with results of the most unequivocal advantage. But as a remedy in the cold stage, very seldom have we ventured on its employment.

Dr. Mackintosh seems to have drawn his experience of its benefits from the trial of the remedy on patients of a robust constitution—generally, soldiers. But in debilitated frames we would most religiously eschew the experiment—preferring safety to novelty of practice in such cases.

The judicious interference of the lancet, in patients of a vigorous constitution, during the interval of the paroxysms, often arrests the intermittent without the further exhibition of quinine—or facilitates the cure under the administration of that excellent antiperiodic tonic.

There are no additional reflections of the author under the head of intermittent that demand notice. There is an obvious incompleteness in his consideration of intermittents from the omission of the best modes of treating the complicated cases, which are of frequent occurrence, in which there exists some visceral lesion, with the protracted continuance of the ague. Besides, there is no reference to these several varieties of intermittent, which we often see in malarian districts; such as the misplaced, the malignant, or comatose, etc.

"Malignant Remittent or Yellow Fever." This is a disease of warm climates, "the milder forms of which depend upon general functional derangement, which runs more quickly into disease of structure than is observed in the fevers of this country. Remittent fever has a very wide range of character; modifications of the complaint occur without end, according to the organ or organs affected, the character of that affection, the constitution and habits of the patient, and the locality of his place of residence. In its severest form, the viscera of the three great cavities are implicated from the first onset of the disease, and there is no complaint in which the appearance on dissection may be so truly predicted." p. 134.

The author coincides with Rush, Johnson, and other high authorities in regarding yellow fever as a more intense form of bilious remittent. The appearances on dissection are analogous, and the most successful mode of treatment is the same in both. Copious detractions of blood are our main dependence in yellow fever, which destroys life very rapidly by inflammation of either the brain, stomach, intestines, liver, spleen, etc., or sometimes by the coincident structural lesion of several of the vital organs. The author condemns emetics and drastic purgatives in yellow fever—and observes in reference to cold affusion, that after the publication of Dr. Currie's work, it became a popular remedy in remittent fevers, "but much mischief followed, and it has fallen into disuse." "The application of blisters, and other counter irritants are highly serviceable after bleeding, etc., but should never be had recourse to in this, or any other fever, in the early stage of the disease." Stimulants of course, with bark and kindred remedies, are decidedly contra indicated. The bowels must be kept freely open by calomel, in moderate doses, aided by castor oil, and other mild cathartic remedies.

"Infantile Remittent."—"Many diseases which occur in infancy and childhood have obtained this name, viz: inflammation of the brain and lungs, the irritation fever produced by teething and worms, rheumatic affections, etc.; in all of which, and even in cerebral and pulmonary inflammations, there are very remarkable remissions in young subjects. But the disease which is to be considered in this section is a febrile affection, which is in general found to depend on irritation, inflammation, or ulceration of the mucous membrane of the stomach and bowels."

The symptoms, as detailed by the author, which mark the presence of infantile remittent are thirst; fretfulness, heat of the skin; hurried breathing; restlessness at night; fetid alvine discharges; or constipation, "the child cries frequently, and draws its knees up to the breast—it cries more when the belly is touched, which is hotter than the rest of the body, and tympanitic." Cold water is eagerly drank, but after a copious draught abdominal pain is induced—the stomach is irritable, and the tongue is occasionally red round its edges; and the hands and feet apt to become cold, whilst the rest of the body is parched, and the face flushed. If the fever continues a few days the brain becomes disturbed in its functional powers—but it is difficult to determine whether or not structural disease has arisen in either the substance or investments of the encephalon. Respiration may be seriously affected in this fever from inflammation of the bronchial membrane. If the fever, in a subdued form, persists a week or two, dropsical effusion may supervene upon the irritation of the abdominal viscera.

Appearances on dissection reveal to us the chief traces of the disease to be abdominal. Sometimes peritoneal inflammation, from an extension of the ulceration from the mucus coat of the intestinal tube to the peritoneum, through the muscular and mucus tissues. The mesenteric glands are found enlarged, and filled with a cheesy looking matter, which, without good foundation, is looked upon generally, as the result of scrofulous inflammation." On cutting into the stomach and bowels, the mucus membrane will be found in various conditions, occasionally very vascular, thickened, softened, or ulcerated.

In the head, effusion in the ventricles, and also between the arachnoid and pia mater, with great vascularity of the latter membrane, are often discovered. In the thorax occasionally are seen evident traces of morbid vascularity in the bronchial lining; the lungs at times being diseased.

It is not an easy matter to discriminate infantile remittent from hydrocephalus internus merely by the symptomatology. We have known instances in which infantile remittent fever was treated for dropsy of the brain. Nor is it less a matter of difficulty in diagnosis to decide between the hydrencephaloid affection, resulting from exhaustion, brought on by purgation and genuine remittent fever. The history of the complaint, with a wary and skilful review of the therapeutical means employed, may lead the practitioner to an enlightened conception of the true pathology of the case.

"Causes."—These are indigestible food, such as crude vegetables, sweet meats, etc.; the habit of allowing children to eat too many articles of food at one meal; together with insufficient clothing, etc., "teething sometimes produces symptoms like those above described."

"Treatment.—Abstinence from solid food is necessary; even biscuits, crusts of bread, and the pulp of oranges, frequently produce relapses."

Leeches and fomentations to the abdomen are demanded. Mild laxatives are required. We have generally preferred one or two grs. of calomel with four or five grs. of rhubarb, or caster oil, frequently repeated.

"Continued Fever."—Passing over two or three pages of verbal and nosological criticism, in which the author indulges towards Dr. Cullen, we will come to what he says concerning continued fever. Under this head he discusses, *first*, fevers from functional derangement; *secondly*, fever from inflammation; *thirdly*, fever from congestion; *fourthly*, a mixed form of fever, consisting of a combination of these three, but in which congestion predominates at last, commonly called typhus and synochus.

The functional form of fever is very liable to attack children; and after what was said by the author under the head of infantile remittent, we can perceive no necessity for what he urges under the title of "fever from functional derangement." The same plan of treatment is available for "fever from functional derangement occuring in children, as for infantile remittent fevers."

"Fever from inflammation is generally ushered in with rigor. The symptoms vary according to the organ principally affected; but in all cases where there is great excitement, the breathing is quick and anxious, the belly costive; the tongue becomes parched; but it may be loaded or very red, with its papillæ much raised, or intensely red only at the tip and round the edges; the pulse is generally full, strong and bounding, beating above a hundred, perhaps even 130 in a minute; there is also oppression at the precordia. "In very acute cases, I have observed the skin not only parched and burning, but red, making a considerable approach towards an exauthematous affection."

Should the head be the seat of the inflammation, there will be delirium, redness of the eyes, starting of the tendons, sometimes convulsions, etc.

The author intends to draw a line of demarcation between those cases of inflammatory fever in which the brain itself is inflamed, and those in which the membranes are in a state of inflammation. But it appears to us that his labored diagnosis is defective, and that the practitioner will constantly find at the bed side that such distinctive symptoms as those laid down with such studied precision by the author, are a fallacious guide in pointing to a just diagnosis of the case. There is not one of them which is entitled in the smallest degree, to the character of pathognomonic.

Inflammatory fever may terminate by hemorrhage from the bowels, or lungs—or by purulent deposits in the subcutaneous cellular tissue, or by profuse sweats. "But these natural terminations are not to be depended on."

If the lungs are affected the respiration will be laborious—there may be cough—with more or less expectoration—the patient has a sense of rawness under the sternum, and a stitch in the side. If the seat of inflammation be within the abdomen—there will be pain on pressure; but it must be remarked, that when the mucus membrane of the intestines is the seat of the phlogosis, frequently little or no pain is experienced even upon pressure. The patient lies on his back with the knees drawn up, that the abdominal parietes may be relaxed; there is more or less tympanitis; and the heat is greater over that part of the body than any other. Nausea and vomiting are more or less severe, the patient incessantly demanding cold water.

"The condition of the tongue, has, I fear, been too much disregarded. The most extensive inflammation, and disorganizations of various kinds, may be going on in the mucus membrane of the stomach and bowels, without producing redness of the tongue or elevation of the papillæ. Nevertheless, when the tongue is in that condition, or when it is covered with small ulcers, or when it looks red and glazed, or as if skinned, with or without patches of white fur, we are enabled to determine that the lining membrane of the alimentary canal is in a diseased condition."

"*Appearances on Dissection.*—It may safely be said that there is not an organ or tissue of the body which has not been seen disorganized in fevers, and particularly in inflammatory fevers." etc. p. 160.

The author does not dwell on these organic lesions, but passes on to the treatment. In the succinct statement of the methodus medendi the author has manifested an enlightened judgment in reference to the most appropriate means of curing inflammatory fever. Bleeding, general and local;—a gentle emetic;—followed by gentle laxatives;—a bland liquid diet;—small doses of the solution of the tartrate of antimony;—perfect quietude of mind and body, with abstraction of all causes of excitement; cold or tepid bathing, local or general as the case may require—these he gives as constituting the best remedies in case of this variety of febrile seizure. He has, we conceive, overlooked the alterant power of mercury—a remedy which in judicious hands will often save the organs from destructive lesion, when no other therapeutic agents will prove available.

"The causes of the failure of bleeding in this, and other diseases, are; *first*, most physicians order the precise quantity of twelve or sixteen ounces of blood to be taken from all adults, without reference to sex, age, peculiarities of constitution, or the actual pathology of the disease. *Secondly*, By the long period which is allowed to elapse between the bleedings, the strength is diminished, while little progress is made in eradicating the disease. *Thirdly*, No difference is in general made between bleeding a plethoric individual, and one who is in the opposite condition of system. *Fourthly*, The period of the disease influences a pathological physician, while it does not one who never looks at the inside of a dead body. *Fifthly*, The good effects of a general bleeding are frequently lost, by not following it up, in proper time, by a second evacuation; or by local bleedings, which are often found to be most efficacious. *Sixthly*, The good effects of bleeding are often marred by neglecting to employ counter stimulation, and counter irritation, as well as by loading the patient with too many bed clothes, and by errors of diet. The patient should be seen within a few hours after the first bleeding, and the operation should be repeated at a short interval, if necessary. If this be done, particularly if followed by laxatives, blisters, and the use of the tartar emetic, it will rarely be necessary in an inflammatory fever, however acute to bleed a third time. But if, at the second or third visit, we find the patient so well as not to require further loss of blood, we are not to conclude that he is out of danger; and it is necessary to impress upon the minds of students and young practitioners, that if they are to do good in such a case, the greatest attention must be paid at the very commencement of the disease: vigilance at this period will save much subsequent trouble and anxiety. When leeches are necessary, they should be applied as near the affected organ as possible. With regard to antimony, objections are very justly entertained against its use, when the stomach and bowels are either irritated or inflamed." p. 162.

Speaking of the employment of stimulants in inflammatory fever the author remarks, that "although I have seen marked benefit produced by stimulants, yet I have more frequently observed mischief; they are most beneficial when exhibited to patients with either a compressible, or a very irritable pulse, and to those who experience profuse perspirations."

"Congestive Fever."—Under this head we find only a meagre account of a variety of fever which the late eloquent Armstrong expatiated on with so much power of argument and illustration, and which Sydenham seemed to have understood. We will not dispute about a mere name, provided the reader do not attach too mechanical a conception to the term, congestion. If by the name congestive fever be only understood, what Sydenham evidently meant, a fever of such intense force of action as to oppress the functions of life, then without detriment to the cause of practical medicine, the term may be retained. But loose phraseology is apt to engender, or at least accompany, a loose and inaccurate style of thought. We think that Dr. Rush has given a better name to such a variety of fever, which he entitled "suffocated." Dr. Mackintosh is very unsatisfactory in his discussion of this variety of fever—both in his pathological and therapeutical reflections.

"Typhus and Synochus Fevers."—"In the disease which is now to be sketched, there is a combination of the last three described fevers, appearing under two forms:

1. The functional fever, subsequently united with congestion, and this forms, I apprehend, the typhus of authors.

2. The inflammatory fever, subsequently united with congestion, and this is the synochus of authors.

We cannot admit the correctness of our author's grounds of difference in the above two forms of febrile affection. Typhus fever is *ab origine*, as frequently complicated with local inflammations as Synochus is, and we might affirm, with the utmost confidence, that agreeably to the patient and minute researches of M. M. Louis and Chomel, typhus is more liable to be complicated with lesions of nutrition—or processes of destructive vascular action than any other variety of fever. Death may not cut off the patient in so rapid a manner in typhus as in yellow fever—but the fatal catastrophe is more frequently connected with lesions of nutrition in typhus than in yellow fever. Lesions of circulation—violent and rapid inroads on the nervous and vascular systems—occur in biliou and especially in yellow fever—but the disease does not leave such palpable demonstrations of deep structural alteration as are revealed in the examinations of the bodies of those dying of typhus. The author has been misled in this matter by theoretic refinement. We refer to the able work of Louis for ample proof of the above remark.

The author, very properly, insists strenuously on abstaining from stimulants in the first stage of typhus, and in reference to emetics, which are now *out of fashion* with many physicians—(for there is a fashion in medicine far as potent sway as in matters of costume) he says, "emetics cannot be too highly extolled in the last stage of some cases of fever, particularly the varieties called typhus and synochus, but only in those in which the bronchial tubes become filled with muco-purrulent matter. This happens in consequence of the patient being too long asleep, or not coughing up the matter before too much has been secreted." p. 170.

There are some excellent general reflections, under this head, upon fever in general, which we shall transcribe. The American editor has given some valuable suggestions, which we regret our limits forbid transcribing.

"Cleanliness, free ventilation, and quietness, are three great and essential circumstances to be attended to in the treatment of fever. The alvine evacuations should be removed instantly out of the room; and it is of great consequence to attend that the quantity of bed-clothes be not too great in the first and second stages of fever when the skin is parched, or too small when the patient is approaching to the state of collapse. The extremities should be examined at every visit by the physician as sometimes the symptoms are aggravated in consequence of cold limbs, which will require no other remedy than the application of heat."

Why is it that we have such "discrepant histories of fever;" and why is it that such "opposite practices have been recommended to our notice." The author gives the following explanation of these "glorious uncertainties of medicine."

1st. A difference in the character of the prevailing epidemics, and the constitutions of the persons affected; for example a functional fever will bear stimulating remedies which would kill a person laboring under an inflammatory fever, particularly if the inflammation affected a vital organ. A stimulant given in congestive fever may operate beneficially; whereas in functional fever, or in inflammatory fever, it would be very injurious. A well fed, and previously healthy soldier, who has no cares, will in general have a high toned-fever; whereas a poor, ill fed, and badly clothed laboring man, worn out by cares and anxieties, and living in an ill-ventilated and filthy apartment, will be affected with one of an opposite character.

2nd. An arbitrary and too often empirical practice, which has hitherto been too frequently followed. One physician always bleeds in every case of fever, another stimulates, and while the results are analysed, perhaps it will be found that the proportion of deaths is the same, and even these will vary to support the one practice or the other according to the habits and constitutions of the patients; for instance, if our army and navy surgeons were to stimulate through the course of the fevers they have to deal with, they would scarcely save a patient; and if practitioners entrusted with the care of the sick poor were to bleed all their cases of fever, they would be quite as unsuccesful.

3d. Writers are too often guilty of an error which all medical men are liable to commit, viz: of mixing up opinions with matters of fact in their statements.

4th. The prevailing habit of drawing sweeping conclusions from one or two facts.

5th. Unphilosophical attempts to bolster up erroneous views by special pleading." p. 172.

We preterm it the brief notice taken by the author of hectic fever, as he promises to illustrate the subject in a subsequent part of his work.

"General Pathology of Eruptive Fevers."—"According to the humoral pathology, the fever is produced by a concoction of the humors by which a precant matter is thrown to the surface, forming the eruption. Other pathologists look upon these diseases as peculiar and essential affections of the epidermis, sometimes *followed* by inflammation of the chest and its accompanying fever; and they account for the sore throat which occasionally occurs by its continuity between the skin and the diseased internal organs.

"My own opinion is, that the eruption ought to be regarded as a mere symptom of this class of disease." p. 183.

The author conceives, after "a long and patient investigation, comparing the symptoms with the appearances found on dissection, that the mucous membranes are the seat of the disease, the nature of which is inflammation, more or less acute and extensive, and that the part most generally implicated, is the mucous membrane of the lungs, particularly in measles and small pox; while that of the bowels is the part chiefly, if not principally, affected in urticaria, roseola, and miliary fever."

Again, says he, "the eruption, in point of fact, ought to be regarded as a natural blister, acting as a counter-irritant. If it is produced by powers inherent in the constitution, that enable it to remove so much of the diseased action from an internal organ, the functions of which are more immediately necessary to life. In slight cases, I conceive the eruption is in proportion to. if it do not exceed the amount of the internal disease. This may be stated without reference to the quantity of the eruption, except perhaps in small pox. There can be no doubt that the eruptions are produced by inflammation of the cutis, which consequently must take off so much of the determination of blood, and so much of the diseased action from the internal organs."

He considers that this view of the pathology of eruptive fevers is substantiated by 1. "The constitutional commotion and oppression of the whole system, and the morbid changes in the functions of various organs, for many days before the appearance of the eruption. 2. By the relief afforded, in general, after the free developement of the eruption.

3. By the increased suffering and danger which exist when the eruption is deficient, or when its repulsion suddenly and prematurely takes place.

4. By the relief which follows proper treatment; and

5. By the appearances observed on dissection.

All this wears an air of great plausibility about it, but we regard the whole pathological description, as founded upon limited conceptions of the nature of this class of fevers.—What are the proofs adduced, but evidences that the morbid action is localized in the dermoid tissue, and that the close and reciprocal relation of sensation and vascular activity existing between the skin, and internal organs, affords a satisfactory ground for a solution of the subsequent phenomena, which are seen by us in this class febrile affections.

Instead of being merely symptomatical the eruption should be regarded as the true disease, and the irritation and inflammation of the brain, lungs, stomach, etc., which often reveal themselves in the progress of small pox, measles, etc., are but the complications of the original malady.

But we have not time or space afforded to enter upon a detailed examination of what we conceive a very defective scheme of pathological exposition of eruptive fevers. We wish the reader of this review of the work to reflect upon the matter, with careful thought, lest his mind become perplexed by the ingenious argumentation of the author. We have very seldom seen small pox fatal from internal inflammation—what the author terms the *symptom*—proving fatal by destroying the structual integrity of the skin—or inducing death by the extensive irritation and suppuration of the surface—as in the secondary fever. Measles and scarlet fever are more liable than small pox to be complicated with inflammation of the air passages, or brain, and in the way pointed out by the author often terminate existence.

Dissections do not demonstrate the verity of the authors peculiar, or rather Broussaian hypothesis, but only prove that there is not that simplicity in such diseases which such a speculative abstraction would insist on. Under the head of scarlet fever we find nothing very striking or interesting. The author very properly insists on the expediency of blood letting in this inflammatory disease. The remarks of the author on the other eruptive fevers are judicious, though not very satisfactory on account of the brevity with which he discusses the great questions involved in their pathology and treatment.

"Part II."—Diseases of the organs connected with the digestive system. "Under this head are arranged various morbid affections—Difficult Dertition—Difficult Deglutition—Cynanche Tousillaris—Cynanche Pharyngea—Inflammation or Ulceration of the Æsophagus—Indigestion—Gastrodynia—Discharges of Blood from the Stomach and Bowels—Colic—Intestinal Concretions—Worms—Inflammatory Affections of the Organs contained within the Cavity of the Abdomen—Cholera—and Diseases of the Liver and Spleen. We pass by several of the first named diseases, as there is nothing in the notice of them of much interest—and briefly analyse what is offered under the head of Indigestion. Under the designation indigestion, dyspepsia, with its usual attendants, flatulency, tympanitis, heartburn, and pyrosis, and also the painful affection termed gastrodynia, are treated.

The author thinks that the physician, to be able effectually to conduct the treatment of dyspepsia should have suffered from it himself. This is abundantly puerile—for although a recollection of his own dyspeptic sufferings may make him very sympathetizing, yet this very state of mind will interfere with a judicious application of rigid means of cure. It would be as sound philosophy to urge that to conduct the parturient process the doctor ought previously have been brought to bed himself! The symptoms of dyspepsia vary—flatulency produces great distress and the escape of the pent up air *sorsum vel deorsum*, affords much relief. The plan of treatment should not be of the routine character, but vary with the case, etc. The causes may be very different—Dr. Wilson has written an excellent work on the subject—he divides the disease into three stages. This arrangement the author objects to, yet he adopts it. Regimen including exercise, clothing, etc., and laxatives are the points to be chiefly attended to in the management of the dyspeptic. Animal diet, according to W. Barras, is the best remedy for gastralgia, which may be mistaken for inflammation of the mucous tissue of the stomach.

Discharge of blood from the stomach and bowels may originate from mere exudation or from erosion, by ulceration, of some arterial or venous radicle. In one case, a child, the blood was found upon dissection, to emanate from a ruptured artery in a cavern in the superior lobe of the left lung—a fistulous opening was found running upwards from this cavern, and communicated high up in the æsophagus.

"The pernicious habit of taking a book or newspaper to read in the water closet, when at stool is very frequently a cause of piles." p. 244.

Piles are often the source of hemorrhage from the rectum etc.

The treatment is obvious—rest—mild laxatives—astringents, etc. Sometimes it is necessary to apply caustic to arrest bleeding from the anus.

Colic is very succinctly treated by the author. Under the head of Painter's Colic are some very excellent suggestions. We shall transcribe some of his thoughts. The principal symptom is "pain about the umbilicus and pit of the stomach:—at first it is dull and remitting, but gradually increases to be severe and constant. The pain, in some severe cases, strikes through the back, and patients have told me that it resembled a stab through the body; others have felt as if they were cut in two at the umbilicus. In other cases the pain extends to the arms and hands, down the back and pelvis often affecting the lower extremities. The integuments of the abdomen feel retracted and hard, and I have seen the strongest men rolling and weeping like children." Vomiting of mucus may occur to the slight relief of the patient. The pulse, is at the beginning, unaffected by any marked deviation of the normal state; "in the end, however, it becomes quick and small. It has been remarked by some, that the feet and toes are occasionally affected, as in gout. "Spontaneous relief is said to follow a copious discharge of scybalous matter, like sheep's droppings, mixed with mucus and considerable quantities of blood. Sometimes the disease produces palsy of the superior extremities, and occasionally it terminates in death, which is preceded by a loss of sight and hearing, delirium, and convulsions." p. 248.

Appearances on dissections are redness, thickness, and ulceration of the mucous membrane of the alimentary canal, and often enlargement of the mesenteric glands, corresponding to the inflamed or ulcerated portions of this membrane. "The redness varied from that bright rose, even to violet and brown, it was disposed in points, in streaks and in patches, and sometimes occupied an extent of several feet. The thickness was variable. The ulcerations were founded almost always toward the termination of the small intestines, near the valve of the colon, which was sometimes destroyed; and in cases where diarrhœa prevailed, ulcerations were founded in the colon; and sometimes they were observed in the stomach. They were occasionally deep and numerous; sometimes the stomach and intestines were perforated."

*Treatment.*—General and local bleeding, counter-irritation over the stomach; calomel and opium given in pills containing four or five grains of each, repeated at short intervals, so as to affect the system as speedily as possible.

Turpentine, by the mouth and by injections, and applied through flannel over the belly, croton oil, given during the administration of the calomel and opium, are to be used. The mercury must touch the gums before the patient is safe—and therefore the calomel, we think, should be given in larger doses, than those recommended by the author. We find nothing of moment under the heads, respectively laid down by the author, till we come to his general remarks, relating to inflammatory affections of the organs contained within the cavity of the abdomen.

The symptoms evincive of the presence of inflammation in the abdominal viscera are not always correspondent to the intensity of the morbid action. The pain upon pressure in cases of peritonitis, we know to be intense, but when the mucous coat of the intestinal canal is inflamed there may be little or no pain on pressure made by the hand on the abdominal covering. There are two terms much and mischievously employed in medical nomenclature to which the author objects, as being liable to a loose and inaccurate application. These words are sympathy and debility. Vagueness of expression we should ever avoid in a physical science, such as medicine is. Sympathy has, very often, no precise pathological sense attached to its too facile use.

"I have, says Dr. Mackintosh, frequently seen it highly injurious in practice, thus, in inflammation of the stomach and bowels, I have seen the most deadly cerebral symptoms lighted up, which were not treated, because it was supposed the affection of the brain was only *sympathetic*, not real. I have seen the same thing happen in fevers, gout, rheumatism, etc." p. 264.

In speaking of acute peritonitis he states, that, according to the experience derived from his observations, bleeding, both general and topical, should be had recourse to; in very slight cases, we may trust to local bleeding by leeches, but when the inflammation is severe, the lancet should be used to such an extent as the nature of the case demands, so as to make a decided impression upon the disease, and upon the system." As a cathartic he prefers two grains of calomel, and six of rhubarb, and repeated every three hours. Blisters, late in the disease may be used, the abdomen should be completely covered, when the blister is necessary. Digitalis he has no confidence in. Where there exists any distension of the abdomen from tympanitis, we have an admirable remedy in turpentine by injection, in the proportion of half an ounce, or an ounce, in eight or ten ounces of gruel, or it may be put into a weak tobacco injection.

Puerperal Peritonitis, or puerperal fever, is a very fatal malady, and requires the most energetic interference of the physician to save from death the patients committed to his charge. The celebrated Dr. William Hunter, who gave brandy in the disease, saved only one patient out of thirty-two. Dr. Leake, employed bleeding in small quantities, and at long intervals; he gave his patients bark, beef tea, and cordials, to prevent putridity, and he lost thirteen out of nineteen patients in one season.

Mr. Sley, of Leeds, saved only three out of thirteen cases, before he began to bleed; under an improved mode of practice, copious bleeding and purging, he lost only two out of thirty-six patients.

The pathology of puerperal peritonitis is essentially inflammatory; there can be no room for rational doubt on this point, after the revelations of truth which have taken place in late years on this question. Neither the pain, nor pulse, can be taken as a guide; the pain is often severe, but sometimes it is slight, and the pulse is small, quick, and frequent, though hard. The lochia and milk are suppressed, and the bowels are found in a variable state, but more frequently than otherwise, constipated. But the knife has made known to us the severe inflammatory nature of the disease—the peritoneum is diffusively inflamed, or deep marked patches of redness are seen on it, sometimes the bowels are glued together by the organizable lymph thrown out, and very often a sero-gelatinous fluid, or thick curdy matter is found in the cavity of the abdomen. The treatment must be heroic, the lancet, leeches from 25 to 200 applied to the abdomen at one time, purgative, and blisters; constitute the most available means of arresting the rapid disorganizing action going on. Dr. Armstrong gave 20 grains of calomel, with one or two grains opium—if given directly after a full bleeding. The author has given some useful practical suggestions on chronic peritonitis, which we are unable to copy.

"Inflammation of the mucus membrane of stomach and bowels. It is a point of the first importance to determine the natural condition of the mucus membrane, in order to enable us to ascertain the appearances produced by disease. It is admitted, I believe, by every one, that the mucus membrane of the stomach and bowels, in the most healthy state in which we see it after death, has a whitish appearance, with a slight tint of rose color; that although blood vessels may be seen here and there, yet they are not observed arborescing in great numbers, nor do we see any discolored patches, unless there has been some great impediment to the circulation, or they have been produced by a natural change towards decay. Indeed, it is to be apprehended that some of the tints described with so much minuteness and accuracy by French pathologists, may be attributed to this last cause. It is stated that the stomach becomes more vascular and of a redder color, during the act of digestion, than at any other period, which appears to be very probable, and may account for the red appearance found in the bodies of criminals after execution." The splenic portion of the stomach seems most liable to inflammatien. The diseased appearances of the stomach may be divided into *color*, *vascularity*, exudation, *alterations of structure.* We are very liable to deception respecting the color of the gastric mucus tissue. Every change of color is by no means to be attributed to inflammation. Dr. Yellowly has conclusively shown that an intense degree of redness may be seen after death in the mucus tissue of the stomach, where no suspicion could attach of previous disease in that organ. The principal discolorations of the mucus membrane resulting from disease are bright red, dark red approaching to purple, brown, slate colored and black. In considering how far mere vascularity is the sequel of morbid action, previously existing in the mucus coat, we must try to ascertain whether it is arterial or venous; whether it is a hyperamia which has occurred in the struggles of death, or a fulness of the vessels arising from gravitation. Exudation merits careful attention with regard to tenacity, quantity, and color. If it is viscid, and in considerable quantity, upon a surface which presents many red vessels, it may be regarded as the product of irritation and inflammation. It varies much in color, sometimes of a red aspect, sometimes white, etc.

Alterations of structure are denoted by pulpiness, with thickening of the mucus membrane, and ulcerations of various kinds and degrees. The inferior half of the ileum is more frequently found ulcerated than any portion of the alimentary tube. Ulceration is frequently the result of acute phlogosis of the mucus tissue. We must refer our readers to the author for a more expanded illustration and enforcement of the subject, and likewise to the works of Broussais, Andral, Craigie and others.

We find that we must hurry on in our review else w hall trespass too much on the patience of the reader. Let us see what the author says on bowel complaints of children.

"In the course of practice, it is distressing to see so many children carried to the grave from a diseased condition of the alimentary canal; although there is no class of complaints, which, when taken early, and treated according to good pathological principles, are more under control. They frequently terminate by producing marasmus, and the complaint which I have presently to notice, under the name tabes mesenterica." p. 297.

The author condemns in severe terms, the practice so generally adopted after the birth of a child—that of forcing down—wantonly," as he expresses it—the throat of the poor innocent—a dose of castor oil, or sugar and water, or molasses, "for the unnecessary purpose of purging away the dark matter which collects in the large intestines during the last two or three months of the uterine life."

"Not long ago, I was called to see a child under a fortnight old, who was taking half a grain of calomel and two of scammony, twice a day, although it had from fifteen to twenty stools during the course of twenty-four hours, notwithstanding the exhibition of occasional doses of chalk mixture. In such cases, the drastic purgatives are given in the first instance to "clear out the bowels," and afterwards persevered in "to improve the evacuations." Another fruitful source of bowel complaint in children is improper, or too much food. Too often the infant is forced to take panada, or tea made of bread or cracker, or thin gruel—the consequence is flatulency, to relieve which, Dalby's carminative is given, then castor oil to relieve the costiveness induced by the opiate, and thus the poor creature is dragged into a protracted torturing process, springing from ignorance and indomitable prejudice.

An opposite course is to be pursued, nothing but milk is to be allowed for diet, and a little sweet oil given, should the bowels be constipated. We have known infants destroyed by the exhibition of syrup of rheubarb, calcined magnesia, sulphur, and such remedies. We pass on to diseases of the organs connected with the respiratory system. After some general remarks well worthy attentive perusal by the student, the author divides diseases of the respiratory organs into those which affect the mucus membrane of the air passages, such as bronchitis, laryngitis, croup, and hooping cough, and those affecting the parenchymatous part of the respiratory system. Andral, deservedly, receives a high eulogy from our author, against whose methods of ascertaining the states of the organs contained within the chest, a great deal of opposition has been made, and many frivolous objections urged, principally by three classes of practitioners. First, Those who are too well employed, and who have not time to learn any thing new. Secondly, Those who are dull of hearing, or devoid of the power of discrimination between sounds which have some resemblance to each other. Thirdly, Those who are too indolent or too old." There is in the author's general remarks on the stethoscope a spirit of severe criticism against those who refuse to bear witness to its value. His language is occasionally rather tinctured with coarseness of rebuke—a fault which pervades many parts of the work. But his reflections on this point are deserving of a serious and earnest perusal by all who desire to grow in professional attainments.

Pretermitting a good deal of valuable matter under the respective heads named above, we shall present some of the author's views and statements on various subjects, which we take from the second volume.

"Part. IV.—Diseases of the Circulating System."—After several pages devoted to the consideration of the sounds of the heart, as evinced in health and in diseased states of that organ, the author thus speaks of the causes of diseases of the heart. "The causes of diseases of the heart are imperfectly known; affections of the lungs, which give to long continued and severe dyspnœa, are, no doubt, among the most frequent causes, they are considered by Laennec as the best ascertained. We know, perhaps with more certainty, that diseases of the heart give rise to various affections of the lungs, more particularly hæmoptysis and pulmonary apoplexy."

Every cause which disturbs the balance of the circulation, producing an overload of blood about the heart and lungs, excites this class of affections; hence I have been able to trace it to long continued intermittent fevers. It would appear that rheumatism is of frequent cause of enlargement of the heart; it is well known by practical men, that pericarditis sometimes come on during an attack of acute rheumatism. We find that those who have suffered repeatedly from acute rheumatism, not unfrequently fall victims to enlargement, or other diseases of the heart."

"Angina Pectoris.—This dreadful disease generally makes its attack in the following manner. It is commonly first felt when an individual is walking up hill; he is suddenly and unexpectedly seized with an agonizing sensation in his breast, a little to the left of the sternum; he experiences a sense of constriction and suffocation, which obliges him to stop. After a little rest, these symptoms disappear, and he flatters himself that it is nothing more than a common stitch in the side, from walking too quickly. I have known a person fall down in a state of temporary asphyxia, even on the first attack, etc." p. 21.

The author appears to lean towards the opinion of Dr. Hosack, that "a disproportionate accumulation of blood in the heart and large vessels;" gives rise to the seizure. We believe that an attack of Augina Pectoris may spring from neuralgia of the heart—ossification of its valves or large vessels, or hypertrophy of the organ. The treatment consists in a careful regulation of diet and exercise of the patient, alterant doses of calomol or blue pill; counter-irritation by tarter emetic, or an issue.

The author has some judicious reflections under the respective heads of Pericarditis, Carditis, Hypertrophy of the Heart, etc., which we are compelled for want of space to omit any analysis of, but we will notice a disease of pretty frequent occurrence, compared to the above named affections—it is Phlegmasia Dolens. Dr. Davis' opinion, derived from dissections that this affection springs from inflammation of the veins the author regards as not only plausible but just; at least, from his observations he has been conducted to the conclusion that no reasonable mind can reject Dr. Davis' pathology, although in the present state of our knowledge, he is far from alledging that inflammation of the veins is the only cause of this affection. Phlegmasia may occur during pregnancy, but most frequently it comes on after delivery. Sir Astley Cooper and Dr. Dease, have seen cases of the disease from tying the saphena vein in men. The treatment is antiphlogistic—bleeding general and topical—blisters over the lower part of the abdomen and groin of the affected side; tartar emetic internally administered, calomel and opium, fomentations over the lower part of the abdomen, and finally a bandage.

"General remarks on Diseases of the Brain.—After a rapid notice of the physiological experiments and inquiries conducted during the few last years into the anatomy and functions of the brain, the author speaks of the principal symptoms which are supposed to indicate disease of the brain.

1. *Headache.*—This symptom depends on very opposite cases, plethora, and anæmia, stimulant drinks and diet, and depletion, fasting and eating, all may cause it.

2. *Vertigo.*—This symptom likewise has its origen in very opposite states of the nervous and vascular systems.

3. *Convulsions.*—Sometimes indicate disease of the brain. They frequently attend inflammation, and occasionally depend on organic lesions. Excessive vascularity of the cerebral vessels will cause convulsions, and so will a deficient and sudden depletion of those vessels.

4. *Rigidity of the extremities.*—The occurrence of this symptom is rarely wanting in *ramollissement*, or softening of the brain.

5. *Coma.*—This alarming symptom, may depend on opposite states of the encephalon.

6. *Fever.*—Is by no means a certain accompaniment of inflammation of the brain, there may be meningitis or cerebritis, without fever.

7. *Delirium.*—Is a very general consequence of inflammation of the brain. But it may exist without inflammation, and inflammation may exist without delirium.

8. *Paralysis.*—Is a very frequent result of inflammatory diseases within the skull, and of tumours and apoplexy.

Dr. Abercrombie's position, that "no material change can take place in the absolute quantity of blood circulating in the vessels of the brain," is successfully controverted by the author, and the practical deductions springing from this physiological speculation are proved to be dangerous.

But we must stay our hand of exegetical remark on Dr. Mackintosh's valuable work, and hope that from the analysis presented, our readers will purchase and read the entire production. His style is often slovenly and sometimes inaccurate, and the condensed mode pursued by the author in the discussions of many important questions leaves the mind unsatisfied as to the solution of the points in controversy. With these, and several other minor abatements, it is still a work of great utility, and although we prefer the last edition of Good's Study of Medicine by Cooper, as a more classical, learned, and ornate work, we conclude by again recommending Macintosh's Practice of Physic, as a safe and judicious guide in the practice of medicine. J. P. H.

